# Supplementary material for: Control Effects of Short-Term Heatwaves on a Holocyclic Aphid
Source: Insects. 2024 Feb 1;15(2):100. doi: 10.3390/insects15020100 (PMC10888736; doi:10.3390/insects15020100)
Supplement: Supplementary file 1 [file insects-15-00100-s001.zip › insects-2793753-supplementary.pdf]

Table S1. Proportion (Mean ± SE) of different morphs deposited by *A. glycines* on day 1, exposed to diurnal 35°C for 7 days begin at different developmental stages

Initial stage of aphid exposed to diurnal 35°C	Proportion of *A. glycines* morphs in offspring (%)		
	Virginoparae	Gynoparae	Male
1st–stadium	67.43 ± 0.09 a	32.57 ± 0.09 a	0
2nd–stadium	75.66 ± 0.15 a	24.34 ± 0.15 a	0
3rd–stadium	74.95 ± 0.06 a	25.05 ± 0.06 a	0
4th–stadium	82.66 ± 0.06 a	17.34 ± 0.06 a	0
Adult stage	86.56 ± 0.02 a	13.44 ± 0.02 a	0
Aphids reared at constant 20°C (CK)	84.33 ± 0.01 a	15.67 ± 0.01 a	0

Means within a column followed by the same letter do not differ significantly (HSD test, $P < 0.05$).

Table S2. Proportion (Mean ± SE) of different morphs deposited by *A. glycines* on day 7, which were exposed to diurnal 35°C for seven days begin at different developmental stages

Initial stage of aphid exposed to diurnal 35°C	Proportion of *A. glycines* morphs in offspring (%)		
	Virginoparae	Gynoparae	Male
1st - stadium	19.29 ± 0.05 a	80.71 ± 0.05 a	0
2nd - stadium	28.61 ± 0.08 a	71.39 ± 0.08 a	0
3rd - stadium	26.17 ± 0.06 a	73.83 ± 0.06 a	0
4th - stadium	27.52 ± 0.06 a	72.48 ± 0.06 a	0
Adult stage	23.30 ± 0.07 a	76.70 ± 0.07 a	0
Aphid reared at constant	21.77 ± 0.02 a	78.23 ± 0.02 a	0

Means within a column followed by same letter do not differ significantly (HSD test, $P < 0.05$).

Table S3. Proportion (Mean ± SE) of different morphs deposited by *A. glycines* on day 13, which were exposed to diurnal 35℃ for seven days begin at different developmental stages

Initial stage of aphid exposed to diurnal 35℃	Proportion of *A. glycines* morphs in offspring (%)		
	Virginoparae	Gynoparae	Male
1st －stadium	0	33.97 ± 0.10 a	66.03 ± 0.10 b
2nd －stadium	0	43.08 ± 0.17 a	56.92 ± 0.17 b
3rd －stadium	0	39.75 ± 0.17 a	60.25 ± 0.17 b
4th －stadium	0	67.50 ± 0.14 a	32.50 ± 0.14 b
Adult stage	0	75.71 ± 0.03 a	24.29 ± 0.03 b
Aphid reared at constant 20℃ (CK)	0	0 b	100 a

Means within a column followed by same letter do not differ significantly (HSD test, $P < 0.05$)
